# The Role of Birds of the Family Corvidae in Transmitting *Sarcocystis* Protozoan Parasites

**DOI:** 10.3390/ani11113258

**Published:** 2021-11-14

**Authors:** Evelina Juozaitytė-Ngugu, Saulius Švažas, Donatas Šneideris, Eglė Rudaitytė-Lukošienė, Dalius Butkauskas, Petras Prakas

**Affiliations:** Nature Research Centre, Akademijos Str. 2, LT-08412 Vilnius, Lithuania; evelina.ngugu@gamtc.lt (E.J.-N.); saulius.svazas@gamtc.lt (S.Š.); donatas.sneideris@gamtc.lt (D.Š.); egle.rudaityte@gamtc.lt (E.R.-L.); dalius.butkauskas@gamtc.lt (D.B.)

**Keywords:** *Sarcocystis*, corvids, definitive host, intermediate host, ITS1, *cox1*, molecular identification

## Abstract

**Simple Summary:**

Members of the genus *Sarcocystis* are protozoan parasites that infect mammals, birds, and reptiles. *Sarcocystis* spp. have an obligatory two-host prey-predator life cycle. Sarcocysts form in the muscles and central nervous system of the intermediate host, while oocysts and sporocysts develop in the small intestine of the definitive host. There is a lack of studies on omnivorous birds of family Corvidae as potential definitive hosts of *Sarcocystis* spp. Until now, only *S*. *ovalis* has been confirmed to be transmitted via corvids. In the current study, 91 small intestine samples from six corvid species from Lithuania were examined for the presence of *Sarcocystis* spp. that use birds, carnivorous mammals, and cervids as intermediate hosts. Oocysts of *Sarcocystis* spp. were observed in 43 samples (47.3%) using a light microscope. Based on molecular methods, 11 *Sarcocystis* spp., (*S*. *columbae*, *S*. *cornixi*, *S*. *halieti*, *S*. *kutkienae*, *S*. *lari*, *S*. *turdusi*, *S*. *wobeseri*, *S*. *arctica*, *S*. *lutrae*, *S*. *ovalis*, and *S*. *oviformis*) were identified. These results indicate that corvids may transmit some species of *Sarcocystis* that use birds and mammals as intermediate hosts.

**Abstract:**

Members of the family Corvidae are ecologically flexible omnivorous birds, particularly adaptive to urban habitats, and living in proximity to humans; these birds may serve as definitive hosts (DH) for *Sarcocystis* spp., but research about this is lacking. In the present study, intestinal samples from 91 corvids collected in Lithuania were molecularly tested by species-specific PCR targeting the ITS1 and *cox1* genes and subsequently sequenced for the presence of *Sarcocystis* spp. Under a light microscope, oocysts of *Sarcocystis* spp. were observed in 43 samples (47.3%), while molecular methods, detected *Sarcocystis* spp. in 77 birds (84.6%). Eleven *Sarcocystis* spp. (*S*. *columbae*, *S*. *cornixi*, potentially pathogenic *S*. *halieti*, *S*. *kutkienae*, *S*. *lari*, *S*. *turdusi*, *S*. *wobeseri*, *S*. *arctica*, *S*. *lutrae*, *S*. *ovalis*, and *S*. *oviformis*) were identified in the intestinal samples from six corvid species from Lithuania. Infections with multiple *Sarcocystis* spp. were detected in 79.2% of the infected corvid birds. Three of the identified *Sarcocystis* spp. use corvids as intermediate hosts (IH); therefore, corvids may serve as IH and DH of the same *Sarcocystis* species. Based on molecular results and on corvid diet, omnivorous corvids may play an important role in transmitting *Sarcocystis* spp.

## 1. Introduction

Representatives of the genus *Sarcocystis* (Apicomplexa: Sarcocystidae) are parasitic protozoa widespread in reptiles, birds, and mammals. They are characterised by an obligatory prey-predator two-host life cycle [1]. Asexual multiplication with formation of sarcocysts occurs in extra-intestinal tissues, mainly the muscles, of the intermediate host (IH), while sexual stages of the parasite’s life cycle (oocysts and sporocysts) develop in the small intestine of the definitive host (DH) [2]. The sarcocyst structure is one of the most important criteria for describing *Sarcocystis* spp., and species cannot be distinguished according to the morphology of parasite sexual stages observed in the DH [1].

Birds have been shown to be DH of at least 17 *Sarcocystis* spp. worldwide [1,2,3,4,5,6,7,8]. Birds of prey have been reported as DH of *Sarcocystis* spp. that mainly use birds and small mammals (rodents, lagomorphs, etc.) as IH [1,8,9,10]. The role of omnivorous birds in transmitting *Sarcocystis* spp. is unclear; however, based on phylogenetic studies, omnivorous birds are likely to be involved in transmitting several *Sarcocystis* spp. that use carnivores and ungulates as IH [11,12,13]. 

Corvidae is a family of ecologically flexible omnivorous birds, particularly adaptive to urban habitats close to humans [14,15,16,17]. Carrion of mammals and birds constitutes a significant part of the corvid diet, particularly of common ravens (*Corvus corax*) and hooded crows (*C*. *cornix*) [14,18]. Based on their diet, corvids may act as DH of *Sarcocystis* spp. [19], but they have received little attention [4,20]. Currently, only one *Sarcocystis* species, *S*. *ovalis*, is confirmed to be transmitted by corvids [20,21]. This species employs cervids such as moose (*Alces alces*), red deer (*Cervus elaphus*) and sika deer (*C*. *nippon*) as IH [22,23,24] and common magpie (*Pica pica*) [21] and Japanese jungle crow (*C*. *macrorhynchos*) as DH [20].

The aim of the current study was to molecularly identify *Sarcocystis* spp. in intestinal samples from six corvid species from Lithuania. 

## 2. Materials and Methods

### 2.1. Animal Collection and Oocysts/Sporocysts Isolation

A total of 91 birds from the Corvidae family were collected between 2015 and 2021. All birds were found dead (as a result of collisions with motor vehicles, power lines, buildings, etc.) and obtained from the Kaunas T. Ivanauskas Zoology Museum, the Lithuanian national authority responsible for monitoring dead or wounded wild birds. Bird samples were kept frozen at −20 °C until a microscopic examination had been conducted. Intestinal samples of 33 hooded crows, 25 common ravens, 21 western jackdaws (*Coloeus monedula*), 5 rooks (*Corvus frugilegus*), 4 common magpies and 3 Eurasian jays (*Garrulus glandarius*) were examined for *Sarcocystis* spp. Oocysts/sporocysts of *Sarcocystis* spp. were isolated from the intestinal mucosa of each bird using previously described methodology [25]. All samples underwent further molecular analysis, regardless of whether oocysts/sporocysts were visible under a light microscope.

### 2.2. Molecular Analysis

Sixteen *Sarcocystis* spp. with birds as confirmed or presumed DH were tested for whether they could be found in the intestinal samples of corvids from Lithuania (Table 1). Ten of these species, (*S*. *calchasi*, *S*. *columbae*, *S*. *cornixi*, *S*. *corvusi*, *S*. *fulicae*, *S*. *halieti*, *S*. *lari*, *S*. *turdusi*, *S*. *wobeseri*, and *S*. *kutkienae*) are found in the muscles of birds as IH; two species, (*S*. *arctica*, and *S*. *lutrae*) use carnivorous mammals as IH; and four remaining species (*S*. *frondea*, *S*. *hardangeri*, *S*. *ovalis* and *S*. *oviformis*) employ cervids as IH [1,11,12,26,27].

Approximately 200 µL of intestinal sediment was taken from each sample and prepared for DNA extraction using GeneJET Genomic DNA Purification Kit (Thermo Fisher Scientific Baltics, Vilnius, Lithuania). Nested PCR (nPCR) was used to amplify DNA fragments of all parasite species in the study. The external primers SU1F/5.8SR2 amplified the internal transcribed spacer 1 (ITS1) region in *Sarcocystis* spp. that use birds and carnivores as IH [28]. The SF1/SR5 primer pair was applied for the amplification of partial cytochrome c oxidase subunit I (*cox1*) gene of *Sarcocystis* spp. that use cervids as IH [29]. Internal nPCR primers, which were developed and used in this study, are listed in Table 1. PCR reactions were conducted using DreamTaq PCR Master Mix (Thermo Fisher Scientific Baltics, Vilnius, Lithuania) according to the manufacturer’s protocol. The PCR cycling conditions were as previously described [25], with modified annealing temperatures (57–65 °C) depending on the primer pairs used. PCR products were visualised, purified, and directly sequenced as previously described [30]. Nucleotide BLAST was used to compare the obtained sequences [31]. The sequences generated in the present study are available in GenBank with Acc. No. OK481182–OK481382.

### 2.3. Statistical Analysis 

The statistical analyses were performed with Quantitative Parasitology 3.0 software [32]. The Sterne’s exact method was used to calculate 95% confidence interval (CI) for prevalence [33]. To compare *Sarcocystis* spp. detection rates in the analysed host species, it was estimated the overall frequency of positive parasite cases, which was calculated as the ratio of positive cases from the host species sample size multiplied by the 16 *Sarcocystis* species tested. The unconditional exact test, which is more sensitive in detecting differences, especially in small sample sizes, evaluated differences in the prevalence of the detected *Sarcocystis* spp. and in the frequency of positive parasite cases [34].

## 3. Results

### 3.1. Sarcocystis spp. Identification in Intestine Samples of Birds of Family Corvidae

Microscopic examination detected, *Sarcocystis* spp. oocysts in 47.3% (43/91) of birds examined (Table 2). Oocysts measuring 20.4 × 19.3 μm (12.3–25.8 × 12.2–23.7 μm; *n* = 69) were seen under a light microscope in intestinal mucosa, while free sporocysts were not detected. The molecular method detected *Sarcocystis* spp. 1.8 times more often (77/91, 84.6%), which was significantly higher (*p* < 0.001) than the microscopic method. In four cases (two hooded crows and two western jackdaws), *Sarcocystis* spp. were observed microscopically, but species-specific nPCR did not detect amplified DNA fragments of parasites. Molecular examination revealed high *Sarcocystis* spp. rates in examined corvid species, varying from 66.7% (in western jackdaw and Eurasian jay) to 100% (in common raven and common magpie). Comparing *Sarcocystis* spp. infection prevalence obtained in three corvid species with adequate sample size to draw reliable conclusions (*N* > 20), molecular analysis detected significantly lower prevalence (*p* = 0.0014) in western jackdaw (14/21, 66.7%) than in common raven (25/25, 100%).

All amplified samples were sequenced and identified as belonging to the species for which the species-specific primers were designed. Comparing ITS1 or *cox1* sequences generated in the current study revealed the presence of 11 out of 16 examined *Sarcocystis* spp. (*S*. *columbae*, *S*. *cornixi*, *S*. *halieti*, *S*. *kutkienae*, *S*. *lari*, *S*. *turdusi*, *S*. *wobeseri*, *S*. *arctica*, *S*. *lutrae*, *S*. *ovalis,* and *S*. *oviformis*) (Table S1). Sequence similarity values were calculated for obtained isolates, within species (sequences obtained during the current study were compared to sequences of the same species available in NCBI GenBank) and, in comparison, to the most closely related *Sarcocystis* species (Table 3). Detected *Sarcocystis* spp. were reliably identified, since sequence similarity within specific species did not overlap in values compared to other valid *Sarcocystis* species. Analysing ITS1 sequences, the highest intraspecific genetic differences were estimated for *S*. *halieti* (0–3.5%) and *S*. *lutrae* (0–4.6%). The GenBank accession numbers of analysed sequences of *Sarcocystis* spp. listed in Table 2 are provided in the supplementary material (Table S2).

### 3.2. Distribution of Sarcocystis spp. in Examined Hosts

All 11 *Sarcocystis* spp. identified in this study were detected in hooded crow samples (Table 4). Nine *Sarcocystis* spp. were found in common raven, six in common magpie, five in western jackdaw and three each in rook and Eurasian jay. *Sarcocystis wobeseri* (*n* = 51), *S*. *halieti* (*n* = 48) and *S*. *kutkienae* (*n* = 45), were detected more often than other examined species (Table 4). *Sarcocystis cornixi* (*n* = 16), *S*. *lari* (*n* = 13) and *S*. *turdusi* (*n* = 13) were detected slightly less, and other *Sarcocystis* spp. were identified in no more than in five birds. *Sarcocystis* spp. that use carnivores and cervids as IH were detected only in hooded crow and common raven; however, the number of investigated individuals of other host species was lower, except for western jackdaw (*n* = 21). When comparing the frequency of positive parasite cases (Table 4) between three corvid species (hooded crow, common raven, and western jackdaw) with adequate sample size to draw reliable conclusions, hooded crow (85/528, 16.1%, *p* = 0.0054) and common raven (60/400, 15.0%, *p* = 0.0254) had significantly higher infection rates than western jackdaw (32/336, 9.5%).

### 3.3. Sarcocystis spp. Mixed Infections

**Figure 1:**
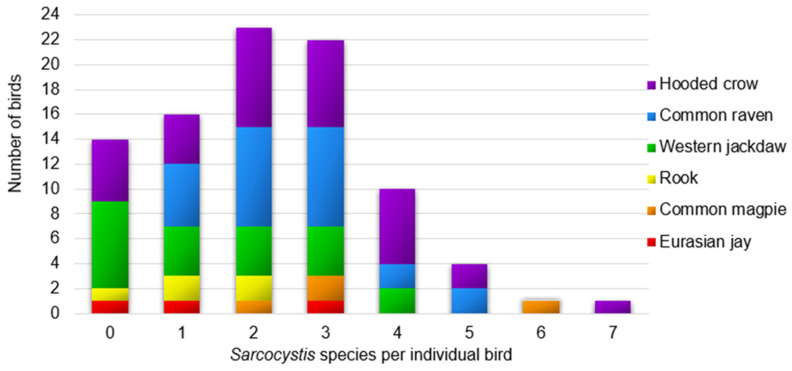
Distribution of the number of *Sarcocystis* species detected in one sample.

Overall, 84.6% (77/91) of examined corvid birds were positive for at least one *Sarcocystis* spp. Multiple species of *Sarcocystis* were common in all corvid host species (Figure 1). Infection with multiple species were found in 79.2% (61/77) of the infected corvid birds, with usually two to four *Sarcocystis* species in one sample. Six bird each had more than four *Sarcocystis* species, and a single common magpie and hooded crow contained the most diverse *Sarcocystis* species, with six and seven parasite species, respectively.

## 4. Discussion

### 4.1. Differences in Sarcocystis spp. Detection Using Microscopic and Molecular Methods

Microscopic analysis is essential to describe and characterize *Sarcocystis* spp. in IH, since parasite species cannot be differentiated by the stages found in DH (oocyst or sporocyst) [1]. Whereas molecular methods can help to identify *Sarcocystis* spp. from intestinal and faecal samples of predators or omnivorous animals [7,8,10,20,21,25,35,36]. However, detection of *Sarcocystis* spp. DNA from intestinal and/or faecal samples does not conclusively prove the role of tested animal as DH of these parasites. The detected DNA may also belong to *Sarcocystis* spp. that had been present in the carrion the bird has been feeding on without infecting analysed animal species. Thus, life cycle experiments are necessary to confirm the results obtained [1]. In the current study, 11 *Sarcocystis* spp. were identified in mucosal scrapings of corvids based on species-specific nPCR targeting ITS1 or *cox1* and subsequent sequencing (Table 3). The overall *Sarcocystis* spp. detection rate was significantly higher (*p* < 0.001) by molecular methods (84.6%) than by microscopic examination (47.3%) (Table 2). The study’s findings are consistent with previous research [25,36], showing that molecular methods should be used to examine all samples, rather than just those that are microscopically positive.

### 4.2. Corvids as Possible DH of some Sarcocystis spp. That Use these Birds as IH

Representatives of the genus *Sarcocystis* have a diheteroxenous life cycle, meaning different animal species serve as IH and DH [1]. However, some *Sarcocystis* spp. that use mice, rats and lizards as IH (*S*. *cymruensis*, *S*. *dugesii*, *S*. *gallotiae*, *S*. *muris*, *S*. *simonyi*, and *S*. *stehlinii*) have been shown to have both diheteroxenous and dihomoxenous life cycles, allowing transmission via cannibalism [37,38,39,40,41,42]. Corvids are known to be IH of three *Sarcocystis* spp. identified in the present study, (*S*. *cornixi* [43], *S*. *kutkienae* [44] and *S*. *halieti* (MZ707148-49, unpublished data) [10]. *Sarcocystis cornixi* and *S*. *kutkienae* form sarcocysts in muscles of corvids, while *S*. *halieti* is multi-host adapted, employing birds of several different orders as IH [45,46]. Birds of prey are identified DH of *S*. *cornixi* and *S*. *halieti* [8,10], and based on phylogenetic placement, birds are the presumed DH of *S*. *kutkienae* [44]. As mentioned before, the detected DNA may belong to the sarcocyst from IH that was eaten and was present in intestines without infecting the DH. However, the other possibility that corvids may act both as IH and DH for the same *Sarcocystis* spp. is intriguing, and more research is needed to reveal this interesting phenomenon. Our data indicate that corvids can feed on carrion of other birds of the Corvidae family, including their conspecifics. For example, groups of hooded crows were observed feeding on carcasses of hooded crows and rooks (presumably killed by poisoning) in a waste disposal area of Klaipeda, Lithuania in January 2016. In several cases, common ravens were recorded feeding on carrion of rooks and western jackdaws killed by cars in Lithuania.

### 4.3. Occurrence of Sarcocystis Species in the Intestinal Samples of Corvids

In the present study, ten *Sarcocystis* spp. (*S*. *columbae*, *S*. *cornixi*, *S*. *halieti*, *S*. *kutkienae*, *S*. *lari*, *S*. *turdusi*, *S*. *wobeseri*, *S*. *arctica*, *S*. *lutrae* and *S*. *oviformis*) were identified in the intestinal samples of corvids (Table 4) for the first time. It should be noted that DH of *S*. *kutkienae*, *S*. *wobeseri*, *S*. *arctica*, *S*. *lutrae* and *S*. *oviformis* were unknown [11,12,44,47,48]. *Sarcocystis ovalis*, which was detected during the current study, was previously determined in faecal and intestinal mucosa samples of common magpie [21] and intestinal mucosa samples of Japanese jungle crow [20]. Of the *Sarcocystis* spp. identified in this work, *S*. *halieti* is potentially pathogenic. Recently, *S*. *halieti*-associated encephalitis was reported in a juvenile free-ranging little owl (*Athene noctua*) from Germany [49]. In this work, *S*. *halieti* was one of the most commonly detected species; it was confirmed in hooded crows (*n* = 20), in common ravens (*n* = 18), in western jackdaws (*n* = 7) and in common magpies (*n* =3) (Table 4). Thus, corvids are likely to be involved in the transmitting pathogenic *S*. *halieti*. 

In the current study, *Sarcocystis* spp. were identified in all six studied bird species (hooded crow, common raven, western jackdaw, rook, common magpie, and Eurasian jay). Hooded crow and common raven had significantly higher frequency of positive *Sarcocystis* spp. cases than western jackdaw. This observation agrees with diet studies of corvids, since common raven and hooded crow are the main scavengers among corvids in Lithuania [50,51]. Carcasses of dead birds and mammals form an important part of the diet of common raven and hooded crow, particularly at waste disposal areas in the winter [51,52]. Common magpie and rook are also frequently observed feeding on carrion of birds [18,51]. Western jackdaws have been observed feeding on small parts of carrion remnants at a waste disposal area in Klaipeda, Lithuania in heavy winter, while Eurasian jays were frequently observed in forests eating the smallest parts of muscles and intestine left in carrion previously used by larger corvids or birds of prey. Based on available data on the corvid diet of and the results of this study, corvids may play an important role in transmitting *Sarcocystis* spp.

Oocysts or sporocysts of *S*. *calchasi*, *S*. *corvusi*, *S*. *fulicae*, *S*. *hardangeri,* and *S*. *frondea* were not detected in corvids in this study. Pathogenic *S*. *calchasi* infects birds of several orders and has been detected in Germany [53,54], USA [55,56], and Japan [57]. This species is also transmitted by *Accipiter* hawks [53,54] prevalent in Lithuania [50,52]; however, *S*. *calchasi* has not been confirmed in Lithuania yet, may be explained by the possibility that corvids are not the DH of this parasite, or this species is absent or rare in Lithuania. In 2013, *S*. *corvusi* was described in the leg muscles of two migrating jackdaws from Lithuania [58], and afterward, there were no more records on this species. Therefore, we hypothesise that this species is absent or rare in the area under investigation. Sarcocysts of *S*. *fulicae* were identified solely in muscles of Eurasian coot (*Fulica atra*) [30], a water bird rarely occurring on land; therefore, corvids have less access to carrion of this species, except for dead individuals rarely found on frozen water bodies in winter [50,51,52]. *Sarcocystis hardangeri* was identified only in cervids from specific regions of Norway and Iceland [23,59], so, this species is unlikely to be distributed in regions south of Norway. Out of five cervid species examined in Lithuania, *S*. *frondea* was found only in introduced sika deer [26]. Therefore, we suppose that *S*. *frondea* was not observed in examined corvids due to this species’ low prevalence in Lithuania.

### 4.4. Molecular Identification of Sarcocystis spp. in Naturally Infected DH

One DH can harbour multiple *Sarcocystis* species [1,8,10,25]. The present study revealed mixed *Sarcocystis* spp. infections in 79.2% of the infected corvid birds. The mixed infections in intestinal or faecal samples of naturally infected wild animals cause issues with identifying *Sarcocystis* spp. In this work, species-specific PCR was applied to detect *Sarcocystis* spp. Despite the high *Sarcocystis* spp. detection rate (86.0%), identification of species is sure to be limited. For instance, four samples were microscopically positive for *Sarcocystis* spp., but molecular analysis did not identify the examined species. These results indicate the presence of other *Sarcocystis* spp. not tested in the intestinal samples of corvids. Other authors have applied methods apart from species-specific PCR, such as cloning, and metabarcoding, to detect *Sarcocystis* spp. from samples containing oocysts or sporocysts [60,61,62,63]. However, previously conducted studies were also limited and revealed only a partial diversity of *Sarcocystis* spp. in certain hosts [8,10,36]. In summary, more sensitive molecular identification techniques of *Sarcocystis* spp. in intestines and faecal samples of omnivorous animals and predators should be further developed.

## 5. Conclusions

Based on species-specific nPCR targeting ITS1 or *cox1* and subsequent sequencing 11 *Sarcocystis* spp. that employ birds (*S*. *columbae*, *S*. *cornixi*, *S*. *halieti*, *S*. *kutkienae*, *S*. *lari*, *S*. *turdusi* and *S*. *wobeseri*), cervids (*S*. *ovalis* and *S*. *oviformis*) and carnivorous mammals (*S*. *arctica* and *S*. *lutrae*) as their IH were identified in the intestinal samples of six corvid species. Ten of these *Sarcocystis* species were confirmed in corvid intestines for the first time. Therefore, the present study’s results indicate that widespread omnivorous corvids, which live close to humans, could be involved in transmitting these *Sarcocystis* spp., including potentially pathogenic *S*. *halieti*. However, the question remains whether corvids are DH of these identified *Sarcocystis* spp. or the detected DNA was a residue of food particles present in the intestine of tested birds.

In four cases, light microscopy detected oocysts of *Sarcocystis* spp. that were not identified by molecular methods. Several other studies also reported that molecular methods were not efficient in revealing the full diversity of *Sarcocystis* spp. in the intestinal and faecal samples of predators or omnivorous animals. Thus, more sensitive *Sarcocystis* spp. identification techniques from naturally infected DH must be developed.

## Figures and Tables

**Table 1:** The internal primers used for the nPCR of selected *Sarcocystis* spp.

Species	Internal Primers		Product Size (bp)
	Name	Sequence (5′-3′)	
*S*. *calchasi*	GsScalF	ATGAACTGCTTTTTCTTCCTCCATT	508
	GsScalR	GACCGTTCAAATATGCTCTTCTTCT	
*S*. *columbae*	GsScolF	ATATGTTCATCCTTTCGTAGCGTTG	579
	GsScolR	GCCATCCCTTTTTCTAAGAGAAGTC	
*S*. *cornixi*	GsScornF2	AGTTGTTGACGTTCGTGAGGTC	483
	GsScornR2	ACACACTACTCATTATCTCCTACTCCT	
*S*. *corvusi*	GsScovF	TATTCATTCTTTCGGTAGTGTTGAG	524
	GsScovR	TTACTCTTTTAACAGCTTCGCTGAG	
*S*. *fulicae*	GsSfulF	CAAAGATGAAGAAGGTATATACGTGAA	449
	GsSfulR	CTTTACTCTTGAAGAACGACGTTGA	
*S*. *halieti*	GsShalF	GATAATTGACTTTACGCGCCATTAC	644
	GsShalR2	CCATCCCTTTTTCTAAAGGAGGTC	
*S*. *kutkienae*	GsSkutkF2	ACACACGGTCGAGTTGATATGAC	625
	GsSkutkR2	TCTTTACCCTTAAACAATTTCGTTG	
*S*. *lari*	GsSlarF	TTCGTGAGGTTATTATCATTGTGCT	545
	GsSlarR	GGCGATAGAAATCAAAGCAGTAGTA	
*S*. *turdusi*	GsSturF	GATTTTTGATGTCCGTTGAAGTTAT	561
	GsSturR	CATTCAAATATGCTCTCTTCCTTCT	
*S*. *wobeseri*	GsSwobF	ATGAACTGCTTTTTCTTCCATCTTT	532
	GsSwobR2	CTCCTCTTGAAGGTGGTCGTGT	
*S*. *arctica*	GsSarcF1	CAAGCACAAATGTATCATCGTCTTA	524
	GsSarcR1	TCCTTTTTATTCTCAAATGACTTCG	
*S*. *lutrae*	GsSlutF1	GAAACGTCTGAAATGATGATGGTAT	528
	GsSlutR1	AAGAGAAAAAGAAAAACAGCCAGAC	
*S*. *frondea*	GsSfroF1	GCTTATTCGATCTGAAATAGCGAGT	495
	GsSfroR1	ATGATGAGCATAACCGCTGTAAATA	
*S*. *hardangeri*	GsSharF1	TTCAATCGTACAATGTGCTCCTTAC	653
	GsSharR2	CCCCAAATACTTGACGACTAGC	
*S*. *ovalis*	GsSovaF2	CTTGCACAGCGTTCTATCTGATTAT	467
	GsSovaR2	CCAAACACTTGTCGAGAACCAAT	
*S*. *oviformis*	GsSoviF2	TGATTGGCGGATTATGTATTTTG	565
	GsSoviR2	ATGTGGTATTTCAAGATGGCTTCC	

**Table 2:** Detection rates of *Sarcocystis* spp. established by microscopic and molecular analyses.

Bird Species	*N*	*Sarcocystis* spp. Positive Animals					
		Microscopy			Molecular Analysis		
		*n*	%	95% CI	*n*	%	95% CI
Hooded crow	33	18	54.5	37.8–71.5	28	84.8 **	68.4–93.8
Common raven	25	16	64.0	43.9–80.4	25	100 **	86.6–100
Western jackdaw	21	7	33.3	15.9–55.1	14	66.7 *	44.9–84.1
Rook	5	0	0	0–50.0	4	80 *	34.3–99.0
Common magpie	4	2	50	9.8–90.2	4	100 ^NS^	47.3–100
Eurasian jay	3	0	0	0–63.2	2	66.7 ^NS^	13.5–98.3
Overall	91	43	47.3	36.9–57.7	77	84.6 ***	75.4–90.8

*N*—number of studied birds; *n*—number of infected birds; * *p* < 0.05, ** *p* < 0.01, *** *p* < 0.001, ^NS^—not significant.

**Table 3:** Identification and genetic variation of detected *Sarcocystis* spp.

Species	Genetic Region	Sequence Similarity %		
		Comparing Sequences of the Same Species Obtained in the Present Study	Comparing Sequences of the Same Species Available in GenBank	Comparing Isolated with Most Closely Related Species *
*S. columbae*	ITS1	98.9–99.8	98.9–99.8	*S. corvusi* 93.0–93.2
*S. cornixi*	ITS1	98.6–100	98.6–100	*S. kutkienae* 89.1–90.3
*S. halieti*	ITS1	98.7–100	96.5–100	*S. columbae* 91.2–92.4
*S. kutkienae*	ITS1	99.0–100	99.0–100	*S. cornixi* 88.4–89.1
*S. lari*	ITS1	99.2–100	98.8–100	*S. jamaicensis* 77.9–78.3
*S. turdusi*	ITS1	99.0–100	98.6–100	*S. kutkienae* 83.6–86.3
*S. wobeseri*	ITS1	99.2–100	99.2–100	*S. calchasi* 91.9–92.7
*S. arctica*	ITS1	100	99.6–100	*S. felis* 88.6–90.0
*S. lutrae*	ITS1	-	95.4–100	*S. canis* 73.2–75.5
*S. ovalis*	*Cox1*	-	98.1–100	*S. hardangeri* 91.7–92.1
*S. oviformis*	*Cox1*	100	99.8–100	*S. ovalis* 89.6–90.6

* Comparison with valid *Sarcocystis* species; - only one sequence was obtained.

**Table 4:** Identification of *Sarcocystis* spp. in examined corvid samples from Lithuania.

Sarcocystis Species	The Family of IH	Host Species						
		Hooded Crow (*n* = 33)	Common Raven (*n* = 25)	Western Jackdaw (*n* = 21)	Rook (*n* = 5)	Common Magpie (*n* = 4)	Eurasian jay (*n* = 3)	Overall (%)
**IH = Aves**								
*S*. *calchasi* *	Cacatuidae; Columbidae; Phalacrocoracidae; Picidae; Psittaculidae	-	-	-	-	-	-	0 (0)
*S*. *columbae*	Columbidae; Laridae	2	2	-	-	-	-	4 (4.4)
*S*. *cornixi*	Corvidae	6	6	-	2	1	1	16 (17.6)
*S*. *corvusi*	Corvidae	-	-	-	-	-	-	0 (0)
*S*. *fulicae*	Rallidae	-	-	-	-	-	-	0 (0)
*S*. *halieti*	Accipitridae; Corvidae;Laridae; Phalacrocoracidae; Strigidae	20	18	7	-	3	-	48 (52.7)
*S*. *kutkienae*	Corvidae	18	11	10	1	3	2	45 (49.4)
*S*. *lari*	Laridae	4	3	2	3	1	-	13 (14.3)
*S*. *turdusi*	Turdidae, Muscicapidae	4	3	4	-	2	-	13 (14.3)
*S*. *wobeseri*	Accipitridae; Anatidae; Laridae	25	12	9	-	4	1	51 (56)
IH = Carnivora								
*S*. *arctica*	Canidae	3	2	-	-	-	-	5 (5.5)
*S*. *lutrae*	Canidae; Mustelidae	1	-	-	-	-	-	1 (1.1)
IH = Cervidae								
*S*. hardangeri *	Cervidae	-	-	-	-	-	-	0 (0)
*S*. *frondea*	Cervidae	-	-	-	-	-	-	0 (0)
*S*. *ovalis*	Cervidae	1	-	-	-	-	-	1 (1.1)
*S*. *oviformis*	Cervidae	1	3	-	-	-	-	4 (4.4)
Overall (%) **		85 (16.1)	60 (15.0)	32 (9.5)	6 (7.5)	14 (21.9)	4 (8.3)	201 (13.8)

* Positive DNA controls were not available in this study. ** Frequency of positive parasite cases was estimated as the ratio of positive cases from the host species sample size multiplied by the 16 *Sarcocystis* species tested.

## Data Availability

Data supporting the conclusions of this article are included in the article. The sequences generated in the present study were submitted to the GenBank database under accession numbers OK481182–OK481382.

## Supplementary Materials

The following are available online at https://www.mdpi.com/article/10.3390/ani11113258/s1. Table S1: Species of *Sarcocystis* validated in various corvid birds using ITS1 or *cox1*, Table S2: *Sarcocystis* species and GenBank accession numbers of sequences used in comparison analysis (Table 3). Sequences obtained in the present study are in boldface.
