# Supplementary material for: The Role of Birds of the Family Corvidae in Transmitting Sarcocystis Protozoan Parasites
Source: Animals (Basel). 2021 Nov 14;11(11):3258. doi: 10.3390/ani11113258 (PMC8614389; doi:10.3390/ani11113258)
Supplement: Supplementary file 1 [file animals-11-03258-s001.zip › animals-1456044-supplementary.pdf]

Supplementary Material

Table S1. Species of *Sarcocystis* validated in various corvid birds using ITS1 or *cox1*

		IH=Aves										IH=Carnivora		IH=Cervidae			
	Isolate	*S. calchasi*	*S. columbae*	*S. cornixi*	*S. corvusi*	*S. fulicae*	*S. halieti*	*S. kutkienae*	*S. lari*	*S. turdusi*	*S. wobeseri*	*S. arctica*	*S. lutrae*	*S. frondea*	*S. hardangeri*	*S. ovalis*	*S. oviformis*
Common raven	CcLT2			OK481186			OK481202				OK481321						
	CcLT3						OK481203		OK481295			OK481372					
	CcLT4						OK481204			OK481308	OK481322						
	CcLT6										OK481323						
	CcLT10						OK481205					OK481373					OK481379
	CcLT12		OK481182				OK481206		OK481296		OK481324						OK481380
	CcLT15						OK481207	OK481250									
	CcLT16						OK481208										
	CcLT17						OK481209				OK481325		OK481377				
	CcLT18							OK481251			OK481326						
	CcLT19						OK481210	OK481252			OK481327						
	CcLT20							OK481253	OK481297								
	CcLT21			OK481187			OK481211	OK481254		OK481309	OK481328						
	CcLT22			OK481188			OK481212		OK481298		OK481329						
	CcLT23		OK481183				OK481213				OK481330						
	CcLT24			OK481189													
	CcLT25						OK481214			OK481310							
	CcLT26						OK481215	OK481255									
	CcLT27			OK481190													
	CcLT28							OK481256			OK481331						
	CcLT29						OK481216	OK481257		OK481311							

	CcLT30							OK481258									OK481381
	CcLT31			OK481191			OK481217	OK481259			OK481332						
	CcLT32						OK481218	OK481260									
	CcLT33						OK481219										
	CcLT1v						OK481220	OK481261			OK481333	OK481374					
	CcLT2v			OK481192			OK481221	OK481262			OK481334						
	CcLT3v			OK481193			OK481222			OK481312	OK481335	OK481375					
	CcLT4v										OK481336						
	CcLT5v						OK481223	OK481263			OK481337						
	CcLT6v							OK481264			OK481338						
	CcLT7v						OK481224	OK481265			OK481339	OK481376					
	CcLT8v							OK481266			OK481340						
	CcLT9v						OK481225	OK481267			OK481341						
	CcLT10v																
	CcLT11v						OK481226	OK481268			OK481342						
	CcLT12v							OK481269			OK481343						
	CcLT13v						OK481227	OK481270			OK481344						
	CcLT15v		OK481184				OK481228	OK481271	OK481299		OK481345					OK481378	OK481382
	CcLT16v						OK481229	OK481272		OK481313	OK481346						
	CcLT18v		OK481185				OK481230	OK481273	OK481300		OK481347						
	CcLT19v						OK481231				OK481348						
Hooded crow	CcLT20v						OK481232	OK481274			OK481349						

	CcLT21v						OK481233	OK481275			OK481350						
	CcLT22v								OK481301		OK481351						
	CcLT23v																
	CcLT24v			OK481194													
	CcLT25v						OK481234				OK481352						
	CcLT26v						OK481235				OK481353						
	CcLT27v						OK481236	OK481276									
	CcLT28v										OK481354						
	CcLT29v																
	CcLT30v																
	CcLT31v			OK481195				OK481277			OK481355						
	CcLT32v																
	CcLT33v						OK481237										
	CcLT34v			OK481196			OK481238	OK481278			OK481356						
	CcLT35v			OK481197			OK481239			OK481314	OK481357						
Western jackdaw	CmLT1						OK481240	OK481279		OK481315	OK481358						
	CmLT2						OK481241	OK481280			OK481359						
	CmLT3										OK481360						
	CmLT4							OK481281		OK481316	OK481361						
	CmLT5						OK481242	OK481282	OK481302		OK481362						
	CmLT6						OK481243	OK481283			OK481363						
	CmLT7							OK481284		OK481317							
	CmLT8							OK481285			OK481364						
	CmLT9																

	CmLT10																
	CmLT11																
	CmLT12							OK481286									
	CmLT13						OK481244	OK481287									
	CmLT14							OK481288	OK481303	OK481318							
	CmLT15																
	CmLT16																
	CmLT17																
	CmLT18										OK481365						
	CmLT19						OK481245				OK481366						
	CmLT20						OK481246										
	CmLT21																
Rook	CfLT7								OK481304								
	CfLT8			OK481198					OK481305								
	CfLT9			OK481199					OK481306								
	CfLT10																
	CfLT11							OK481289									
Common magpie	PpLT1									OK481319	OK481367						
	PpLT2						OK481247	OK481290			OK481368						
	PpLT3						OK481248	OK481291			OK481369						
	PpLT4			OK481200			OK481249	OK481292	OK481307	OK481320	OK481370						
Eurasian jay	GgLT1			OK481201				OK481293			OK481371						
	GgLT2																
	GgLT3							OK481294									

Table S2. *Sarcocystis* species and GenBank accession numbers of sequences used in comparison analysis (Table 3). Sequences obtained in the present study are in boldface.

Species	GenBank acc. no.
S. arctica	KF601306-KF601311, KX022108-KX022111, KX156837, KY947310-KY947311, MF596262-MF596282, **OK481372-OK481376**
S. calchasi	FJ232948, KC733715-KC733718, KT945021-KT945022
S. canis	MW136927
S. columbae	GU253885, HM125052, MN450338-MN450339, **OK481182-OK481185**
S. cornixi	JF520781, JN256120, MZ707150, **OK481186-OK481201**
S. corvusi	JN256119
S. felis	AY190082, MN508375-MN508379
S. halieti	MF946589-MF946596, MH130209, MN450340-MN450356, MW929599-MW929600, MZ707148-MZ707149, **OK481202-OK481249**
S. hardangeri	KC209625-KC209633
S. jamaicensis	KY994651
S. kutkienae	MT495389-MT495406, MZ707128-MZ707147, **OK481250-OK481294**
S. lari	JQ733510, MF946597-MF946609, MN450357-MN450364, **OK481295-OK481307**
S. lutrae	KM657773-KM657805, MF596261, MG272296-MG272305, MG372108-MG372109, MT036253, **OK481377**
S. ovalis	KC209644-KC209655, KF241355-KF241382, LC481034-LC481035, MF596210-MF596211, MK234168, **OK481378**
S. oviformis	KC209656-KC209661, KF898107-KF898109, MN339334-MN339338, MT242372, **OK481379-OK481382**
S. turdusi	JF975683-JF975685, KJ540164-KJ540167, KT588510, **OK481308-OK481320**
S. wobeseri	GU475111-GU475112, HM159421, JN256121, MN450365-MN450373, **OK481321-OK481371**
